# Understanding digital inclusion through the transformation of services and users: a narrative review and novel framework

**DOI:** 10.1136/bmjdh-2026-000051

**Published:** 2026-04-15

**Authors:** Ian Litchfield, David Shukla, Sheila Greenfield

**Affiliations:** 1 Applied Health Sciences, University of Birmingham, Birmingham, UK; 2 Birmingham Health Partners, Birmingham, UK; 3 Eve Hill Medical Practice, Dudley, UK

**Keywords:** Health Equity, Health Services Research, Patient Involvement

## Abstract

The move towards digitally enabled healthcare offers the opportunity to deliver better care for all. Despite this potential, existing evidence suggests the same widely reported health inequalities that currently impact underserved populations are retained and often exacerbated by digital health services. In an attempt to overcome this digital divide, the related concept of digital inclusion has emerged that describes the policies, commissioning, and service-level actions and interventions necessary to address the issue. However, existing frameworks which have attempted to describe and understand digital inclusion have failed to reflect the evolution of organisations, services and users over time. Understanding inclusivity through the lens of digital transformation offers one way of providing a more accurate reflection of how contextual influences on digital inclusion relating to users, organisational processes and broader policy level interventions evolve temporally. The work presented here describes a novel framework for digital inclusion that combines the recognised elements of the digital divide with the core principles of digital transformation. The framework provides a multilevel understanding of how policy, organisations, communities and individuals combine and interact over time and successfully provides a unified means of developing and delivering more holistic strategies better equipped to address sustained digital inclusion. The framework is supported by a structured narrative review that provides exemplar evidence that underpins its constructs and theoretical position.

**WHAT IS ALREADY KNOWN ON THIS TOPIC**

- The concept of digital inclusion describes the policies, commissioning and service-level actions and interventions necessary to address digital inequalities.
- Previous attempts at exploring digital inclusion have been limited to specific technologies or conditions, failing to reflect broader contextual influences and how the relationships between digital technologies, users and service providers evolve over time.

**WHAT THIS STUDY ADDS**

- This work proposes a novel framework that uses the principles of digital transformation to capture the changes in user expectations and experiences, organisational processes, and policy level interventions necessary for sustainable digital inclusivity.
- The framework is underpinned by a structured narrative review which evidences the constructs of the framework and further informed its design and theoretical position.

**HOW THIS STUDY MIGHT AFFECT RESEARCH, PRACTICE OR POLICY**

- The framework provides a unified means of developing and delivering more holistic strategies better equipped to understand and address the factors contributing to sustainable digital inclusion.

## Introduction

**Figure 1:**
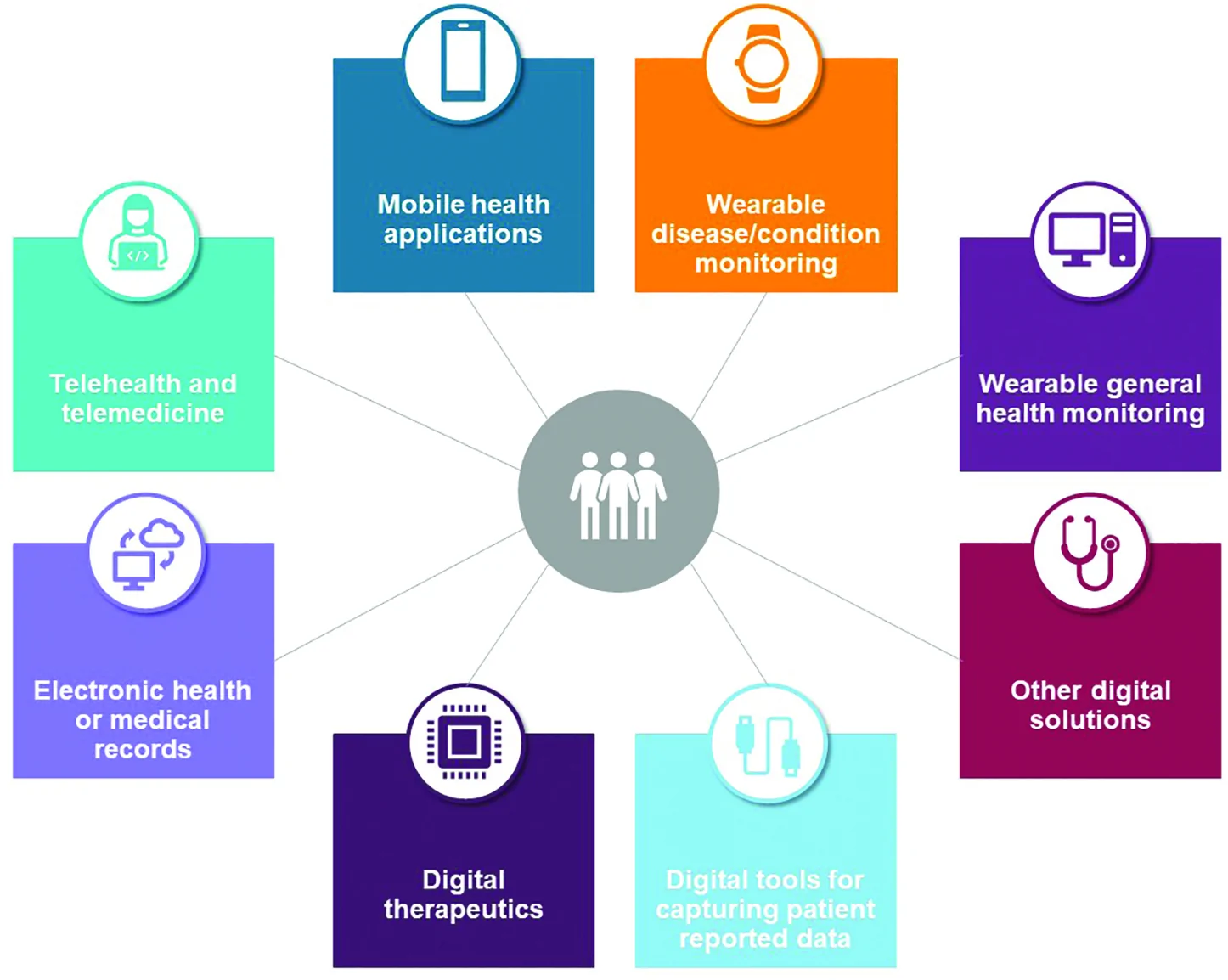
The range and scope of digital health technologies.^3^

The expectation of policymakers and commissioners is that digital health technologies can automate and streamline safe and effective care across the globe,^1^ offering greater opportunity to meet the priorities, preferences and needs of all patients.^2^ Although it appears inevitable in many health systems, such as the UK’s, the move towards digitally enabled healthcare is a complex process involving technologies of varying functionality and purpose (see figure 1),^3^ and incorporating significant changes to pathways, workflows and broader systems of delivery.^4^

Despite the potential of digital health to deliver better care for all, evidence suggests that the same, widely reported health inequalities that impact underserved populations (which are defined here as those who are economically deprived and/or from ethnic minorities that are engaged less effectively by formal healthcare interventions^5^) are retained and often exacerbated by digital health services.^6^ This difference in utilisation of digital healthcare is commonly referred to as the ‘digital divide’^7^ and typically described across three domains: access to digital devices and infrastructure, digital literacy, and the realisation of quantifiable benefits.^8^ In attempting to address this divide, the related concept of digital inclusion has emerged. This goes further to describe the policies, commissioning and service-level actions and interventions necessary to bridge the digital divide. It is predicated on facilitating more equitable access and utilisation of the relevant technology, facilitated by appropriate training and support.^9^

However, previous attempts at understanding digital inclusion have tended to focus on cross-sectional scores to assess the inclusivity of digital health technologies and their impact on specific examples of clinical care.^10^ If senior decision-makers, healthcare providers and researchers are to understand the range of interacting factors that contribute to digital inclusion, then a framework is needed that not only reflects the broader contextual influences of the divide but also how the relationship between digital technology, users and service providers can evolve over time.^11^

One previously successful framework that has enabled a means of understanding the temporal component of the reach and maturity of digital services is that of digital transformation.^12^ It considers technology a driver of transformation but understands how contextual influences of organisational processes, user experience and broader policy level interventions change over time.^12^ It describes the evolution of organisations and users toward digital transformation through three key phases: enablement, where processes and data are made available online; optimisation, primarily describing the necessary training and support for users; and finally, transformation, describing the embedding of digital solutions in routine service delivery.^13^

This work proposes a prototype framework that presents digital inclusion as a function of both the factors contributing to the digital divide and the temporal aspects associated with the digital transformation of a healthcare service. Below, the framework is outlined and described with its central domains and constructs illustrated by examples drawn from a structured narrative review of key evidence.

## Methods

### Study overview

A novel framework is proposed that combines the previously understood concepts of the digital divide with the changes over time described by digital transformation. A structured narrative review was used to produce exemplar evidence to underpin the various approaches, perspectives and experiences of digital inclusion that form the constructs of the framework. This evidence-based approach allowed us to test and develop this framework, further informing its design and theoretical position.

### Outline of framework

**Figure 2:**
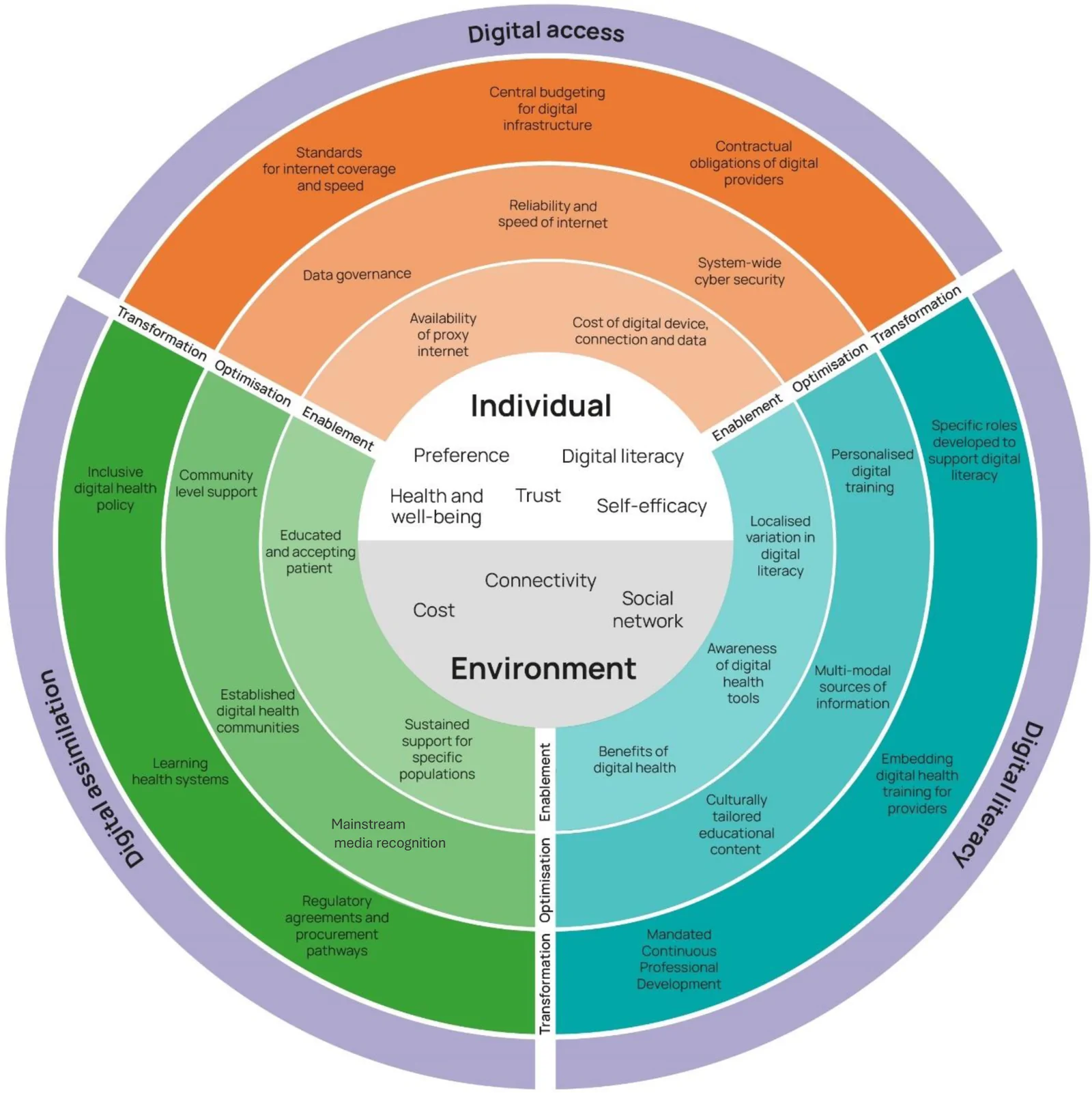
The framework for inclusive digital transformation.

The conceptual framework is presented in figure 2. It is comprised of a core within which the individual is situated and the individual characteristics and contextual factors that can impact their digital inclusion are described. This includes their personal attitudes and beliefs, and their broader environment including socioeconomic status, social network and community resource. Surrounding the central core are three segments each describing an aspect of the digital divide^8^: Digital access; the ability to access the digital technologies; Digital literacy, relating to awareness, education, understanding and demand, and Digital assimilation, relating to the level with which individuals incorporate digital technology into their everyday life.^8^ The three components of the digital divide are further subdivided within each of the three key stages of digital transformation.^12 13^ These begin with the first stage of Enablement; the foundational stage of the digital journey that brings processes and data online towards more automated, technology-enabled interactions. This includes improvement to infrastructure, understanding users’ needs and perceptions, and supporting acceptability of digital healthcare; Optimisation, relating to the factors ensuring security and reliability of digital systems, the training and support required to use digital health services, and the work towards societal understanding and support for digital healthcare; and finally, Transformation, relating to the creation of standards and realisation of long-term financing necessary to maintain and update the infrastructure, the changed role of the workforce, and the policy-driven shifts towards a digitally empowered learning and inclusive health system. The constructs of each sector of the framework were informed and underpinned by the principles of both the digital divide and digital transformation; these were exemplified and refined using the evidence from our structured narrative review. The framework is summarised in figure 2.

### The narrative review

**Figure 3:**
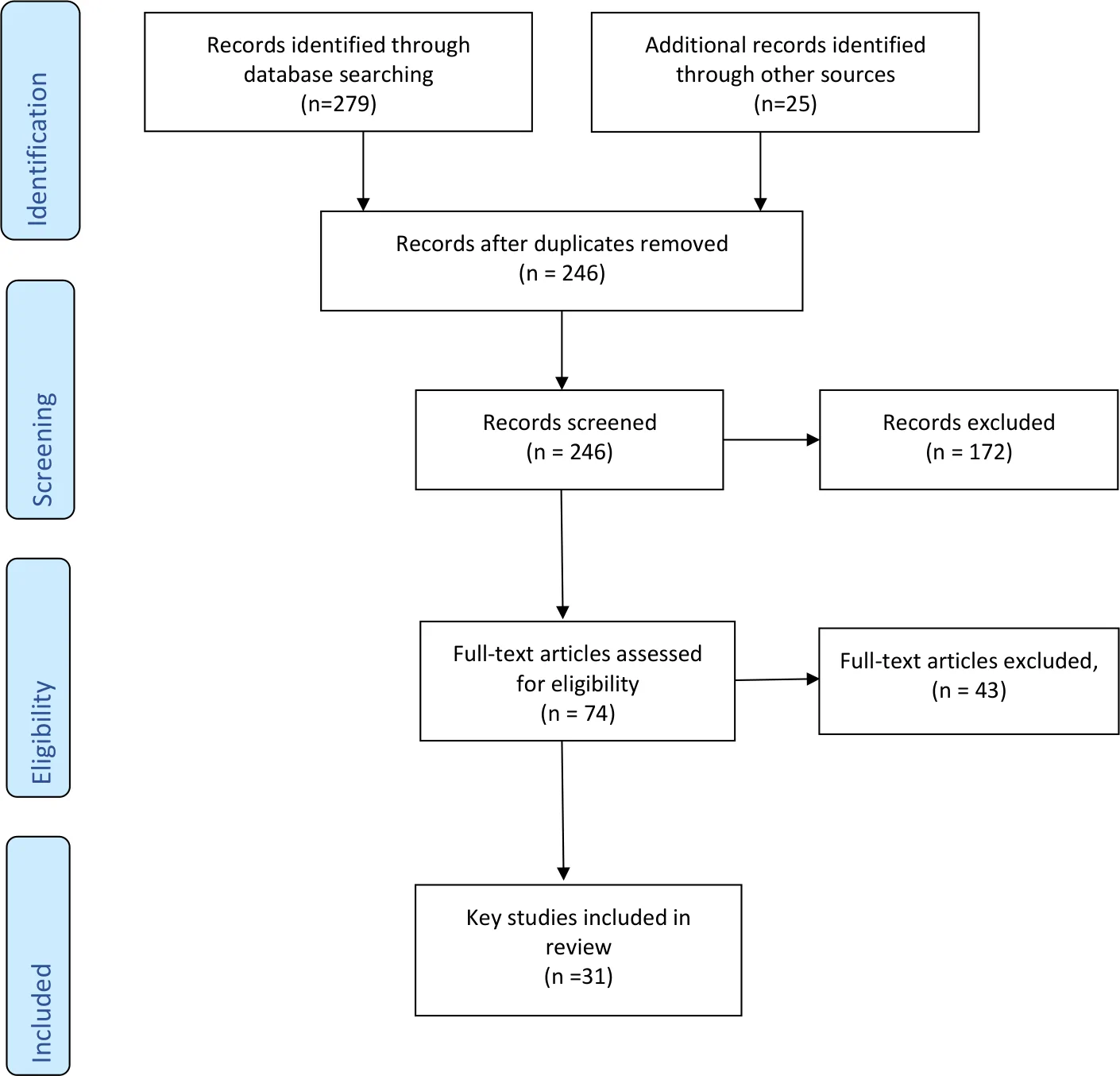
PRISMA diagram. PRISMA, Preferred Reporting Items for Systematic Reviews and Meta-Analyses.

We performed a structured narrative review to illustrate the key concepts and constructs that define the framework. As recommended to improve transparency and demonstrate rigour of narrative reviews,^14^ the process followed the four stages of the Preferred Reporting Items for Systematic Reviews and Meta-Analyses (PRISMA); identification, screening, eligibility and final inclusion, and the search data presented in the PRISMA diagram (see figure 3).^15^

### Inclusion criteria

Study eligibility criteria were established using the Population, Intervention, Comparison, Outcome and Study design framework^16^ (see online supplemental file 1) and we have described our search in accordance with the PRISMA checklist.^15^ The inclusion criteria for our review comprised primary research reviews and systematic reviews complemented by the relevant grey literature including regulatory guidance; only work published in English was considered.

10.1136/bmjdh-2026-000051.supp1 Supplementary data

### Search methods

The literature was searched from January 2015 to June 2025 to determine recent examples and evidence of best practice in digital inclusion. This timespan allows us to describe research relevant to current models of digital health. We created a search for PubMed, supplemented by citation searches and hand searches including those of Google Scholar. In doing so, we used a combination of the search terms including ‘digital inclusion’, ‘underserved populations’ and ‘digital health’. The search terms are described in more detail in online supplemental file 2.

10.1136/bmjdh-2026-000051.supp2 Supplementary data

### Study selection, data extraction and analysis

The first author was primarily responsible for identifying and screening articles by title, abstracts and where appropriate full texts against the inclusion criteria. The criteria for selecting the included work were based on their relevance to the design and delivery of inclusive digital health, with the final selection of papers agreed by all. The data was extracted and consensually located against the various constructs of the framework by all authors. A primarily narrative approach consistent with the recommended analytical method for narrative synthesis was used to summarise the nature and effect of the evidence.^17^

### Patient and public involvement

Patients and the public were not involved in the design or conduct of this review paper.

## Results

We initially retrieved 279 articles. After duplicates and protocols were excluded alongside those removed because they were not specific or relevant to the domains of the framework, a total of 31 papers were included. The PRISMA flow diagram is shown in figure 3. The included papers were authored across a total of 15 countries: 10 in North America (8 in the USA, 2 in Canada), 10 in Europe (including 5 in the UK), with a further 5 authored in Asia, and 3 in Australasia. The selected work included nine systematic reviews, seven narrative reviews, six commentaries by experienced stakeholders and two pieces of primary research. Below we describe the domains and constructs of the framework and their underpinning evidence beginning with the core and working through each sector from digital access through to digital assimilation as summarised in tabular form in online supplemental file 3. A summary table of study characteristics can be found in online supplemental file 4 that includes (1) author and publication date, (2) country of origin, (3) study design and (4) nature of evidence.

10.1136/bmjdh-2026-000051.supp3 Supplementary data

10.1136/bmjdh-2026-000051.supp4 Supplementary data

### The core of the framework

#### Individual and environmental characteristics

Digital inclusion is predicated on a person-centred approach informed or otherwise influenced by an understanding of the individuals’ health and well-being status, their digital literacy, self-efficacy and preferences for care.^18^ Entangled in these preferences are an individual’s age, personality, religion, culture, ethnicity, illness perceptions and, of particular relevance to underserved populations, their experience of mainstream care offers and services.^9^ It is widely ecognised that the ability of any individual to engage with digital health is also shaped by the specifics of their environment. This includes the quality and location of housing stock and availability of the relevant (digital) infrastructure.^19^ It also relates to the extent of their social network, including family or friends, and their ability or willingness to assume a supportive role.^20^

### Digital access

Digital access constitutes the ability to access the necessary hardware, software and internet services associated with utilisation of digital technologies.^8^ This includes the availability, nature, cost and functionality of the digital device, and access to the necessary level of internet connectivity.^21^

### Digital access: enablement

The enablement of digital access is reliant on multiple technical, economic and financial factors. Currently, the cost of digital tools and the data and connectivity related to their access is a major challenge to broader inclusion for many underserved populations, which are often drawn from economically marginalised sections of society.^22^ There are several ways these challenges might be addressed, for example, by providing low-cost or subsidised digital devices, perhaps offered by health services and specific to the condition or care pathway.^18 23^ The use of open source software can also contribute to reducing costs of digital solutions for end users,^24^ as can the provision of internet coverage subsidised by governments or the private sector.^25^

The provision of proxy internet services in local community facilities offers another potential alternative for those struggling for domestic access, with a commonly cited example being the use of public libraries.^26^ However, privacy and security issues of using public Wi-Fi for health-related data remain a concern for many, and such shared spaces are not practicable for those who are housebound or otherwise have difficulties travelling.^26^

### Digital access: optimisation

Optimising digital access requires confidence in the infrastructure’s ability to support digital health services. First, that the reliability and the speed of connectivity meet the expectations and minimum standards needed to support safe and effective clinical care.^27^ Second, in light of longstanding concerns among underserved populations that their data is shared with third parties, including ‘Big Tech’ partners without their prior consent.^28 29^ There are also concerns among patients that their health data is secure from accidental or malicious breaches of system security, particularly in the context of widely reported stories of data breaches in other service sectors (both private and public) including healthcare organisations. To overcome these doubts, it is important that the electronic systems holding the data are demonstrably robust and secure.^30^ This requires messaging that is tailored to specific patient populations and supported by a prescriptive and transparent information governance structure, that recognises the centrality of patients in the decision on who their data is shared with.^29–31^

### Digital access: transformation

The long-term transformation of digital access requires infrastructure funded or coordinated by centralised or governmental organisations. The geographical coverage of existing broadband systems in many high-income countries is inconsistent and particularly patchy for economically deprived urban communities or those in rural locations.^23 32^ It is widely understood that a more coherent approach is needed to fundamentally address these issues.^33^ This involves creating a national infrastructure that is not only robust and pervasive but also capable of linking systems together^33 34^ with contractual obligations placed on providers to meet preagreed standards for coverage, maintenance and improvement.^10^

### Digital literacy

Digital literacy describes the degree of confidence, sophistication and ability with which users are able to use digital technologies.^35^ Below, we consider the importance of understanding the levels of literacy among local underserved populations, the training and support they require, and how the workforce can be empowered to provide that support.

### Digital literacy: enablement

The enablement phase involves understanding the variation in the needs, values and digital experience of local patients and populations,^36^ so that digital health services can meet the requirements of a range of patients.^37^ This involves directing resources towards evaluating patient-specific levels of digital literacy, ideally by using one of the available validated tools.^37^ It is also important to raise awareness of what digital health constitutes and entails among patient populations.^38 39^ Part of this messaging should involve communication around the tangible and specific benefits for patients, such as facilitating improved access to providers or providing support for more successful self-management.^40^

### Digital literacy: optimisation

The optimisation of digital literacy requires provision of the training, educational resources and support necessary to upskill a diverse range of patients. There is growing evidence that the success of interventions supporting digital literacy in patient populations is more successful when tailored to local populations, i.e., reflective of local context and patients’ sociocultural sensitivities and preferences for learning.^39 41 42^ This involves the creation of culturally tailored content eg, using cultural idioms in the description of medical symptoms^43^ or using trainers with the same cultural and linguistic backgrounds.^39^ There is also a role for compiling, and providing access to reliable training resources for independent learning.^44^ There is evidence that healthcare organisations and community-based organisations might be best placed to create these materials due to their closer understanding of the skills needed for specific tools or care processes.^45 46^ The ideal is that the content and mode of the training delivered is tailored to the specific end user with a growing number of examples of personalised training having improved engagement with digital health technologies among underserved populations.^47^

### Digital literacy: transformation

The final phase of digital literacy is transformation which involves organisations ensuring their staff possess the skill sets necessary to implement digital health services for all sections of the patient population. There have been recommendations for the development of specific roles to support digital literacy, including the part played by staff champions to support less digitally literate colleagues,^48^ or to work directly with patients, in newly developed roles such as ‘digital care navigators’.^49^

It is increasingly recognised that the inevitable move toward digital healthcare needs to be reflected in healthcare curricula with all staff routinely trained in digital health technologies and services.^50 51^ There is also recognition of the need for digital health training to become a mandatory element in the continuous professional development of staff,^52^ including secondary and primary care.^53 54^

### Digital assimilation

This describes the degree to which digital technologies are incorporated within usual care to achieve quantifiable outputs.^8^ This includes the acceptance of digital health technologies and practices at an individual and societal level, and their irreversible incorporation into healthcare services through long-term policy decisions.

### Digital assimilation: enablement

The enablement phase of assimilation involves individual patients understanding and accepting digital health services as part of usual care. This requires establishing a rewarding relationship with digital healthcare, whereby patients not only understand and use digital tools but accept and integrate them into the way they access and use healthcare services.^11^ For marginalised and isolated groups, this can include targeted and ongoing initiatives tailored to promote autonomy and independence in specific individuals and groups.^55^

### Digital assimilation: optimisation

Optimisation describes the role communities and social networks play in supporting and normalising the use of digital services. The influence of family, friends and peers on an individual’s health-related behaviour is widely recognised and includes the broader social and cultural acceptance of digital health technologies.^56^ There are examples where community-based interventions such as the introduction of peer supporters^57^ or community-health workers^58^ have sustained engagement and adherence to digital health in underserved populations.^58 59^

There is also a growing role for social media in facilitating engagement with digital health, whether providing advice and support for conditions or specific health needs, or offering informal support for individual tools, with evidence they support increased uptake and dissemination of digital health offers and opportunities.^60 61^ Alongside this is the understanding of the role the news media plays in shaping health and health equity through its ability to frame health agendas, and prime and even persuade the public.^62^

### Digital assimilation: transformation

Transformation requires the implementation of policy that ultimately promotes and enables sustainable inter-agency co-dependence on digital health.^63^ This includes the creation and dissemination of standards and codes to guide the procurement and functionality of technology, such as the interoperability of databases and how digital health technologies might be embedded in usual care.^64–66^

This final level of transformation also incorporates investing in health data science research to further strengthen and embed best practice in digital care. Through the use of real-world data and combined electronic healthcare records, the mature digital health system can learn as it delivers care, incorporating aspects of big data, artificial intelligence, and machine learning.^67 68^

Finally, there are recommendations that digital inclusivity is enshrined in policies that are purposely designed to create an environment that enables secure, inclusive, and sustainable digital transformation, fostering innovation and protecting and reinforcing critical infrastructure.^18 69–71^

## Discussion

### Summary

The potential of digital health to deliver personalised cost-effective care has clear advantages for patients, providers and wider healthcare systems everywhere. However, inequalities in the access and use of digital health persist, with previous attempts at providing a holistic understanding of inclusion failing to accommodate the transformation of users and services over time. The framework presented here contains a more representative architecture that combines the three domains of the digital divide with the temporal evolution of successful digital transformation. Below we look at some of the cross-cutting influences on digital inclusion, their implications for implementation, and suggest some practical steps that might support long-term digital inclusion.

### Strengths and limitations

This novel framework for digital inclusion has served to successfully curate the factors and potential solutions to improve inclusivity. In doing so, it can aid the development of, and comparison across, digital inclusion programmes by explicitly describing the interaction between its various components. However, we acknowledge that the constructs contained within the framework are not necessarily definitive but have the capacity to be modified and extended to incorporate additional constructs and developing evidence.

There are multiple terminologies used across the various literatures sampled in the review and it is acknowledged that terms such as ‘underserved populations’ describe a heterogenous group defined by socioeconomic status, demographic characteristics and broader cultural factors.^5^ The focus of digital inclusion is on bridging the digital divide with underserved, vulnerable or marginalised elements of a population. It can also be reasonably argued that all members of the patient population are vulnerable to digital exclusion as personal or contextual circumstances change, perhaps through illness or the introduction of a new broadband tariff or operating system. This means that understanding and addressing the factors contributing to inclusive digital transformation described by this framework can support a digital health ecosystem that might also benefit more affluent and culturally homogeneous populations.

The universality of the challenges facing inclusive digital health means that although the majority of the exemplar literature was authored by researchers in North America, the geographical spread of authorship demonstrates the global interest and activity in digital inclusion, with many of the barriers identified equally as applicable in low-and-middle-income countries as high-income countries.^72^

To confirm, a narrative review was chosen to illustrate what were frequently well-established constructs of the framework, underpinned as they were by the widely understood concepts of the digital divide and digital transformation. The review was not intended to identify every piece of work relating to every element of the framework (which would have been unnecessarily unwieldy) but instead provide relevant examples of each. It is perhaps notable that for most of the constructs, there was a large evidence base including multiple systematic reviews. However, as we moved towards the final stages of digital assimilation, examples of peer-reviewed literature were far fewer, highlighting the need for more research into the development, delivery and implementation of digital health policy.

### General findings

#### Strategic partnerships

Governments are increasingly understanding the value of investing in measures to address digital inclusion. Key to this is a greater role for strategic partnerships with the private sector and Voluntary, Community, Faith and Social Enterprise organisations (VCFSE).^73^ The cost of digital technologies and infrastructure can render them unaffordable for many underserved populations and publicly funded healthcare models.^74^ For many countries and healthcare systems, this means involving private investment in some aspect of the development or delivery of digital healthcare. Yet despite the need for adaptable and value-based financing mechanisms, the evidence of successful funding models is lacking.^75^ This is due in part to the complexity of attempting to balance the expectations of profit margins of shareholders with the altruism of tax-funded, or hybrid public/privately funded healthcare systems.^76^ However such negotiations play out between commissioners and private partners, their content and outcomes should be transparent, cognisant of the concerns amongst all patients, around governance and ownership of the resulting data.^77^

The involvement of local populations, whether directly or through the VCFSE sector, is needed to authentically frame the development of equitable digital services, including any negotiations with private sector companies.^78^ This is particularly relevant considering the deteriorating trust between underserved populations and mainstream healthcare.^30^ To nurture this relationship, government and healthcare organisations at national and regional level might consider long-term engagement exercises with localised stakeholders. This involves early, sustained and equitable dialogue between commissioners, developers, the private sector and representatives of underserved communities.^78^

#### Research

Research into digital inclusion is a relatively new field, with the majority of primary research conducted in the last ten years, restricted to specific conditions, disciplines and settings, and typically lacking a contextual or policy level perspective.^18^ However, the recognised importance of the interactions between the technology and social, cultural and economic factors on digital inclusion has led to calls for more research to be conducted across academic disciplines and models of healthcare.^40 79^ At the minimum, patient and public involvement should be an inclusive and equitable member of the research team.^80^ One under-explored research methodology that might highlight, and ultimately optimise the considerations for digital inclusivity identified in the framework is community-based participatory research.^81^ Understanding the impact of localised influences on health service delivery, this collaborative approach involves community partners and researchers working together to address health disparities in any given community, recognising the need for the target community to participate in all aspects of the research process in order to enact meaningful change.^81^

#### Co-production

The repeated failure to address the digital divide suggests a fundamentally different approach is needed. One way in which many of the elements that might address digital inclusion can be tailored to better fit local populations is through the process known as co-production.^82^ This encourages service users and those involved in producing or providing a service to identify a problem before empowering them to solve it through open and equitable interaction between all stakeholders.^82^ There is promising evidence of the benefits of co-production in developing digital health solutions.^82 83^ However, the potential conflict between the economic situation of underserved populations, stakeholder-driven needs of the private sector and financial constraints of healthcare organisations and citizen participants can adversely affect the process or the applicability of the solution.^84^ This is perhaps why there are fewer attempts at, or examples of, successful digital health co-production involving underserved groups.^85^

## Conclusions

In summary, digital health equity necessitates a multilevel understanding of how policy, systems, community, individual and intervention factors interact and evolve over time. While many barriers have been identified that contribute to the division in the take-up of digital health, this framework is the first that has attempted to provide a unified means of developing and understanding digital inclusion strategies that recognise the needs of individuals and how organisations and policymakers can better accommodate these over time.
