# Supplementary material for: Understanding digital inclusion through the transformation of services and users: a narrative review and novel framework
Source: BMJ Digit Health Ai. 2026 Apr 15;2(1):e000051. doi: 10.1136/bmjdh-2026-000051 (PMC13492531; doi:10.1136/bmjdh-2026-000051)
Supplement: Supplementary file 1 [file bmjdh-2-1-bmjdh-2026-000051-s001.pdf]

Supplementary File 1 Summary of study eligibility

Type of study	Population or Problem	Intervention or Exposure	Comparison	Outcome
Reviews and primary research grey literature, including commentaries, conference papers, and regulatory guidance	Underserved populations engagement with digital health technologies	Elements or initiatives developed or adapted to improve access, adherence and/or engagement with digital health tools and technologies	Usual or routine care	A range of inclusive or mitigative techniques, approaches, or policies to optimise engagement, uptake, and adherence with digital health tools and technologies.
