# Supplementary material for: Understanding digital inclusion through the transformation of services and users: a narrative review and novel framework
Source: BMJ Digit Health Ai. 2026 Apr 15;2(1):e000051. doi: 10.1136/bmjdh-2026-000051 (PMC13492531; doi:10.1136/bmjdh-2026-000051)
Supplement: Supplementary file 2 [file bmjdh-2-1-bmjdh-2026-000051-s002.pdf]

Supplementary File 2 Sample of search terms

Underserved populations

"vulnerable populations"[MeSH Terms] OR ("vulnerable"[All Fields] AND "populations"[All Fields]) OR "vulnerable populations"[All Fields] OR ("underserved"[All Fields] AND "populations"[All Fields]) OR "underserved populations"[All Fields] OR "medically underserved area"[MeSH Terms] OR ("medically"[All Fields] AND "underserved"[All Fields] AND "area"[All Fields]) OR "medically underserved area"[All Fields]

Digital health

"digital health"[MeSH Terms] OR ("digital"[All Fields] AND "health"[All Fields]) OR "digital health"[All Fields]

Digital inclusion

("digitalisation"[All Fields] OR "digitalised"[All Fields] OR "digitalization"[All Fields] OR "digitalize"[All Fields] OR "digitalized"[All Fields] OR "digitalizer"[All Fields] OR "digitalizing"[All Fields] OR "digitally"[All Fields] OR "digitals"[All Fields] OR "digitization"[All Fields] OR "digitizations"[All Fields] OR "digitize"[All Fields] OR "digitized"[All Fields] OR "digitizer"[All Fields] OR "digitizers"[All Fields] OR "digitizes"[All Fields] OR "digitizing"[All Fields] OR "radiographic image enhancement"[MeSH Terms] OR ("radiographic"[All Fields] AND "image"[All Fields] AND "enhancement"[All Fields]) OR "radiographic image enhancement"[All Fields] OR "digital"[All Fields]) AND ("inclusion bodies"[MeSH Terms] OR ("inclusion"[All Fields] AND "bodies"[All Fields]) OR "inclusion bodies"[All Fields] OR "inclusions"[All Fields] OR "inclusion"[All Fields] OR "inclusive"[All Fields] OR "inclusively"[All Fields] OR "inclusiveness"[All Fields] OR "inclusivity"[All Fields])

Digital divide

"digital divide"[MeSH Terms] OR ("digital"[All Fields] AND "divide"[All Fields]) OR "digital divide"[All Fields]

Digital transformation

("digitalisation"[All Fields] OR "digitalised"[All Fields] OR "digitalization"[All Fields] OR "digitalize"[All Fields] OR "digitalized"[All Fields] OR "digitalizer"[All Fields] OR "digitalizing"[All Fields] OR "digitally"[All Fields] OR "digitals"[All Fields] OR "digitization"[All Fields] OR "digitizations"[All Fields] OR "digitize"[All Fields] OR "digitized"[All Fields] OR "digitizer"[All Fields] OR "digitizers"[All Fields] OR "digitizes"[All Fields] OR "digitizing"[All Fields] OR "radiographic image enhancement"[MeSH Terms] OR ("radiographic"[All Fields] AND "image"[All Fields] AND "enhancement"[All Fields]) OR "radiographic image enhancement"[All Fields] OR "digital"[All Fields]) AND ("transform"[All Fields] OR "transformability"[All Fields] OR "transformable"[All Fields] OR "transformant"[All Fields] OR "transformants"[All Fields] OR "transformation"[All Fields] OR "transformations"[All Fields] OR "transformed"[All Fields] OR "transforms"[All Fields])
