# Supplementary material for: Understanding digital inclusion through the transformation of services and users: a narrative review and novel framework
Source: BMJ Digit Health Ai. 2026 Apr 15;2(1):e000051. doi: 10.1136/bmjdh-2026-000051 (PMC13492531; doi:10.1136/bmjdh-2026-000051)
Supplement: Supplementary file 3 [file bmjdh-2-1-bmjdh-2026-000051-s003.pdf]

Supplementary File 3. Exemplar evidence underpinning framework for digital inclusion

Domain	Level of transformation	Challenges to digital inclusion	Influencing factors
**Digital Access**<br>The ability to access the necessary hardware, software, and internet services associated with utilisation of digital technologies.	**Enablement**<br>Technical, economic, and financial factors relating to digital infrastructure.	Affordability and/or access to hardware, software, and data.	<u>Cost of digital device connection and data</u><br>Low-cost devices for low-resource environments [1]<br>Open source software licensing, subsidies from the private/public sector [2].<br>Cost of connection and/or data e.g. full cost, subsidised, or free [3].
		Availability and access of internet connectivity	<u>Availability of proxy internet</u><br>The use of the internet in safe public spaces such e.g., libraries [4]
	**Optimisation**<br>Patient confidence in digital infrastructure	Perceptions of the reliability and accuracy of digital data captured by tools and monitoring equipment.	<u>Reliability and speed of internet</u><br>The reliability, speed and capacity of broadband access and capacity for all sections of the population should or could be a matter of law and regulation [5].
		The role of third parties and the private sector	<u>Data governance</u><br>Establish digital data governance, data protection, restrict access to patient data by third parties and private sector [6].<br>Ensure patients are involved in decisions on who accesses their data [7].
		Security and confidentiality of individual level health data.	<u>System-wide cyber security</u><br>The use of firewalls and secure servers and other measures to prevent data theft [7]
	**Transformation**<br>Long term (centralised) commitment to	Varying standards across health care systems	<u>Standards for internet coverage</u><br>Standards for connectivity (e.g. coverage and broadband speed) include contractual obligations of providers, reliability in terms of maintenance and improvements [8]

	digital infrastructure	Shifting funding priorities, Short term funding cycles.	<u>Central budgeting for digital infrastructure</u><br>Government grant support for infrastructure, hardware, and software for digital health [8, 9] .
**Digital Literacy**<br>The degree of sophistication with which individuals are able to use digital technologies	**Enablement**<br>Defining and communicating concept of digital healthcare	Variations in patients' values, needs and understanding relevant to digital health technologies	<u>Localised variation in digital literacy</u><br>Consultation/engagement with local patient populations or their ability to use digital health technologies [10]
			<u>Awareness of digital health tools</u><br>Consistent messaging, and accessible information on use and benefits of digital technologies [11, 12].
			<u>Benefits of digital health</u><br>Clear communication on potential benefits [13]
	**Optimisation**<br>Training to meet the needs of patients with a range of numeracy and digital and health literacies.	Inability of patients to use digital health technologies to achieve quantifiable outputs.	<u>Culturally tailored educational content</u><br>Socio-culturally sensitive education and training relevant/suitable to all elements of the local population [12, 14]
			<u>Multi-modal sources of information</u><br>Online sources of training and education for independent learning [15].
			<u>Personalised digital training</u><br>Reflective of patient needs and starting point and related to a broad set of digital skills or specific tools [12] [16]
	**Transformation**<br>Training and support for the routine use of digital tools in delivering care	The lack of adaptation within local workforce and staffing models to support digitally enabled healthcare	<u>Specific roles developed to support digital literacy and healthcare</u><br>Sensitive to a range of patient needs and requirements [17].
			<u>Embedding digital health training for providers</u><br>Digital health training for staff embedded in the curriculum [18]
			<u>Mandated Continuous professional development</u><br>Mandated CPD for digital skills both in staff use of inclusive digital tools but also for supporting patients' use of digital tools [19].

**Digital Assimilation**<br>The degree to which digital technologies are incorporated and utilised in usual care and everyday maintenance of health and well-being	**Enablement**<br>Individual understanding of the value of digital healthcare	The lack of social connections that facilitate an individual's engagement with digital health technologies	<u>Educated and accepting patient</u><br>Promote patient acceptability, and encourage demand for digital healthcare [20]
			<u>Sustained targeted support for specific populations</u><br>Promoting the autonomy and independence of targeted populations e.g., the elderly [21]
	**Optimisation**<br>The support of digital health by (online) community networks	Lack of community or cultural acceptance of digital health technologies	<u>Community level support</u><br>The enrolment of community groups and peer support in the use and acceptance of digital health care [22, 23]
			<u>Established digital health communities</u><br>Online social media networks to support and normalise the use of digital health [24]
			<u>Mainstream media recognition</u><br>The way in which news media shapes (novel) health offers including agenda setting, priming, and persuasion [25]
	**Transformation**<br>Sustained policy driven inter-agency co dependence on digital health	Fragmentary approach to digital health driven by budgetary restrictions, limited capacity, competing organisational priorities	<u>Regulatory agreements and procurement pathways</u><br>Embedding utilisation of digital tools into SOPs, guidelines [26]<br>Formalising procurement pathways, regulatory requirements and interoperability [27, 28]
			<u>Learning health systems</u><br>Including Big data and AI/machine learning [29]
			<u>Inclusive digital health policy</u><br>Universal digital inclusion policy [30, 31]

1. Narayan, S.M., et al., *Access to digital health technologies: personalized framework and global perspectives.* Nature Reviews Cardiology, 2025: p. 1-14.
2. Paton, C., et al., *Open source digital health software for resilient, accessible and equitable healthcare systems.* Yearbook of Medical Informatics, 2022. **31**(01): p. 067-073.
3. Badr, J., A. Motulsky, and J.-L. Denis, *Digital health technologies and inequalities: A scoping review of potential impacts and policy recommendations.* Health Policy, 2024. **146**: p. 105122.
4. Spaulding, E.M., et al., *Leveraging community Wi-Fi and spaces for digital health use.* Front Public Health, 2024. **12**: p. 1418627.
5. Klonoff, D.C., et al., *Digital connectivity: the sixth vital sign.* Journal of Diabetes Science and Technology, 2022. **16**(5): p. 1303-1308.
6. Marcelo, A., et al., *Transforming health systems through good digital health governance.* Manila: Asian Development Bank, 2018.
7. Adjekum, A., A. Blasimme, and E. Vayena, *Elements of trust in digital health systems: scoping review.* Journal of medical Internet research, 2018. **20**(12): p. e11254.
8. Organization, W.H. and I.T. Union, *Digital health platform handbook: building a digital information infrastructure (infostructure) for health*. 2020: World Health Organization.
9. Das, D.K., *Exploring the symbiotic relationship between digital transformation, infrastructure, service delivery, and governance for smart sustainable cities.* Smart Cities, 2024. **7**(2): p. 806-835.
10. Oh, S.S., et al., *Measurement of digital literacy among older adults: Systematic review.* Journal of medical Internet research, 2021. **23**(2): p. e26145.
11. Madanian, S., et al., *Patients' perspectives on digital health tools.* PEC Innovation, 2023. **2**: p. 100171.
12. Whitehead, L., et al., *Barriers to and facilitators of digital health among culturally and linguistically diverse populations: qualitative systematic review.* Journal of medical Internet research, 2023. **25**: p. e42719.
13. Abernethy, A., et al., *The promise of digital health: then, now, and the future.* NAM perspectives, 2022. **2022**: p. 10.31478/202206e.
14. Choudhary, H. and N. Bansal, *Addressing digital divide through digital literacy training programs: A systematic literature review.* Digital Education Review, 2022(41): p. 224-248.
15. Verweel, L., et al., *The effect of digital interventions on related health literacy and skills for individuals living with chronic diseases: A systematic review and meta-analysis.* International Journal of Medical Informatics, 2023. **177**: p. 105114.
16. Wilson, S., et al., *Recommendations to advance digital health equity: a systematic review of qualitative studies.* NPJ digital medicine, 2024. **7**(1): p. 173.
17. Pettersen, S., H. Eide, and A. Berg, *The role of champions in the implementation of technology in healthcare services: a systematic mixed studies review.* BMC Health Services Research, 2024. **24**(1): p. 456.
18. Aungst, T.D. and R. Patel, *Integrating digital health into the curriculum—considerations on the current landscape and future developments.* Journal of medical education and curricular development, 2020. **7**: p. 2382120519901275.
19. Kaihlanen, A.-M., et al., *Continuing education in digital skills for healthcare professionals—mapping of the current situation in EU member states.* International Journal of Health Policy and Management, 2024. **13**: p. 8309.
20. Perski, O. and C.E. Short, *Acceptability of digital health interventions: embracing the complexity.* Translational Behavioral Medicine, 2021. **11**(7): p. 1473-1480.
21. Alruwaili, M.M., M. Shaban, and O.M. Elsayed Ramadan *Digital Health Interventions for Promoting Healthy Aging: A Systematic Review of Adoption Patterns, Efficacy, and User Experience*. Sustainability, 2023. **15**, DOI: 10.3390/su152316503.

22. Poduval, S., et al., *Do community-based digital health inclusion programmes contribute to tackling health inequalities in disadvantaged population groups?: a qualitative study of experiences of a city-wide programme in the North of England.* BMC Public Health, 2025. **25**(1): p. 275.
23. Peng, R., et al., *Unlocking the Power of Peer Support in Digital Use and Digital Health Interventions for Older Adults: A Scoping Review.* Journal of Advanced Nursing, 2025. **n/a**(n/a).
24. Gaddy, A. and J. Topf, *Facebook Groups Can Provide Support for Patients with Rare Diseases and Reveal Truths About the Secret Lives of Patients.* Kidney Int Rep, 2021. **6**(5): p. 1205-1207.
25. Even, D., et al., *News media as a commercial determinant of health.* The Lancet Global Health, 2024. **12**(8): p. e1365-e1369.
26. Marwaha, J.S., et al., *Deploying digital health tools within large, complex health systems: key considerations for adoption and implementation.* npj Digital Medicine, 2022. **5**(1): p. 13.
27. Colloud, S., et al., *Evolving regulatory perspectives on digital health technologies for medicinal product development.* NPJ Digit Med, 2023. **6**(1): p. 56.
28. Bauerly, B.C., et al., *Broadband Access as a Public Health Issue: The Role of Law in Expanding Broadband Access and Connecting Underserved Communities for Better Health Outcomes.* J Law Med Ethics, 2019. **47**(2_suppl): p. 39-42.
29. Sheikh, A., et al., *Health information technology and digital innovation for national learning health and care systems.* The Lancet Digital Health, 2021. **3**(6): p. e383-e396.
30. Marques, I.C. and J.J. Ferreira, *Digital transformation in the area of health: systematic review of 45 years of evolution.* Health and Technology, 2020. **10**(3): p. 575-586.
31. Wagg, S. and B. Simeonova, *A policy-level perspective to tackle rural digital inclusion.* Information Technology & People, 2022. **35**(7): p. 1884-1911.
