# Supplementary material for: Understanding digital inclusion through the transformation of services and users: a narrative review and novel framework
Source: BMJ Digit Health Ai. 2026 Apr 15;2(1):e000051. doi: 10.1136/bmjdh-2026-000051 (PMC13492531; doi:10.1136/bmjdh-2026-000051)
Supplement: Supplementary file 4 [file bmjdh-2-1-bmjdh-2026-000051-s004.pdf]

Supplementary File 4 Characteristics of included studies

Digital divide	***Transformation level***	***Authors (year)***	***Country***	***Evidence type (primary, review etc)***	***Overview of findings***
Access	Enablement	Narayan et al (2025)	USA	Narrative review	Challenges and solutions to digital health technology access
		Paton et al (2022)	International	Rapid review	Exploring impact of open-source coding on cost, accessibility and equitability
		Badr et al (2024)	Canada	Scoping review	Exploring how digital strategies and policies can better support digital equity
		Spaulding (2024)	USA	Narrative review	Challenges and opportunities of using community hosted Wi-Fi to improve digital access/ health
	Optimisation	Klonoff et al (2022)	USA	Narrative review	Connectivity of wireless devices and communication tools for medical care (in diabetes)
		Marcelo et al (2018)	Philippines	Review	Development of a digital health governance framework
		Adjekum et al (2019)	Switzerland	Scoping review	Understand the barriers and facilitators to establishing trust in digital health systems
	Transformation	World Health Organisation (2020)	International	Book	Guidance and recommendations for developing a national digital health system
		Das (2024)	South Africa	Systematic review	Alignment of infrastructure, governance, and service delivery

Digital literacy	Enablement	Oh et al (2021)	South Korea	Systematic review	Evaluate tools assessing digital literacy among older adults
		Madanian et al (2023)	New Zealand	Narrative review	Understand factors influencing patients' engagement with digital health tools.
		Whitehead et al (2023)	Australia	Systematic review	Influences on digital health uptake amongst culturally and linguistically diverse populations.
		Abernathy et al (2022)	United States	Commentary	Review of the promise of digital health and priorities for cross-sector cooperation and collaboration
	Optimisation	Choudhary et al (2022)	India	Systematic review	for marginalised populations in developed and developing countries.
		Verweel et al (2023)	Canada	Systematic review	The effects of e-health interventions on digital health literacy
		Wilson et al (2024)	United Kingdom	Systematic review	Review of outreach or coproduction on inclusive digital health strategies
	Transformation	Pettersen et al (2024)	Norway	Systematic review	Impact of champions as a strategy in inclusive technology implementation
		De Aungst and Patel (2020)	USA	Commentary	Potential educational pathways to prepare health professionals to work with digital tools
		Kaihlanen et al (2024)	Finland	Evidence mapping	Review of evidence describing the existing continuing education in digital skills for healthcare professionals in European Union

Digital assimilation	Enablement	Perski et al (2021)	Australia	Narrative review	Acceptability in digital health research and practice and the influence of social and cultural norms
		Alruwaili et al (2023)	Saudi Arabia	Systematic review	The impact of digital health interventions on healthy, autonomous aging
	Optimisation	Poduval et al (2025)	United Kingdom	Primary research	How community-based digital health inclusion programmes contribute to tackling health inequalities
		Peng et al (2025)	China	Scoping review	Peer support in digital use and digital health interventions for older adults.
		Gaddy and Toof (2021)	USA	Commentary	Use of social media in health advocacy and support
		Even et al (2025)	UK	Commentary	The impact of the news media on various aspects of priming and promoting health innovation
	Transformation	Marwaha et al (2022)	USA	Commentary	Strategies for selecting digital health tools and planning for implementation
		Colloud et al (2020)	Switzerland	Narrative review	Applicability for current regulatory approaches for Digital health technology tools
		Bauerly et al (2019)	USA	Commentary	Explored connection of broadband and telehealth laws with health, making evidence-based decisions to improve health outcomes for all
		Sheikh et al (2021)	United Kingdom	Narrative review	Use of digital health technologies to facilitate a learning health system

		Marquez and Ferreira (2020)	Portugal	Systematic review	Digital transformation of healthcare since 1955
		Wagg and Simeonova (2022)	United Kingdom	Primary research	Impact of policy on digital inclusion in rural areas
